# Bacterial c-di-GMP signaling gene affects mussel larval metamorphosis through outer membrane vesicles and lipopolysaccharides

**DOI:** 10.1038/s41522-024-00508-6

**Published:** 2024-04-04

**Authors:** Xiao-Meng Hu, Lihua Peng, Jingxian Wu, Guanju Wu, Xiao Liang, Jin-Long Yang

**Affiliations:** 1 International Research Center for Marine Biosciences, Ministry of Science and Technology, Shanghai Ocean University, Shanghai, China; 2 Shanghai Collaborative Innovation Center for Cultivating Elite Breeds and Green-Culture of Aquaculture Animals, Shanghai, 201306 China

**Keywords:** Biofilms, Applied microbiology

## Abstract

Biofilms serve as crucial cues for settlement and metamorphosis in marine invertebrates. Within bacterial systems, c-di-GMP functions as a pivotal signaling molecule regulating both biofilm formation and dispersion. However, the molecular mechanism of how c-di-GMP modulates biofilm-induced larval metamorphosis remains elusive. Our study reveals that the deletion of a c-di-GMP related gene in *Pseudoalteromonas marina* led to an increase in the level of bacterial c-di-GMP by knockout technique, and the mutant strain had an enhanced ability to produce more outer membrane vesicles (OMVs) and lipopolysaccharides (LPS). The mutant biofilms had higher induction activity for larval metamorphosis in mussels *Mytilus coruscus*, and OMVs play a major role in the induction activity. We further explored the function of LPS in OMVs. Extracted LPS induced high larval metamorphosis rate, and LPS content were subject to c-di-GMP and LPS-biosynthesis gene. Thus, we postulate that the impact of c-di-GMP on biofilm-induced metamorphosis is mediated through OMVs and LPS.

## Introduction

Biofilms are a prevalent form of bacteria that can colonize various surfaces in the ocean, including rocks, sediments, and the surfaces of plants and animals.^1,2^ They serve essential ecological functions in marine ecosystems,^3^ providing a foundation for microbial life and facilitating the settlement and colonization of other organisms.^2,4^ For instance, biofilms have the ability to induce settlement and metamorphosis in invertebrates,^5–7^ as observed in the case of the mollusc *Mytilus coruscus.*^8–10^ The marine invertebrate initially exists as a planktonic larva and once contacts with biofilms on the substrate, it undergoes alterations in its tissue structure and changes in its habitat, transitioning into a benthic juvenile stage.^11,12^ However, the mechanisms underlying biofilms’ impact on inducing settlement and metamorphosis in invertebrate larvae remain elusive.

Biofilm is composed of bacteria and extracellular polymeric substances (EPS),^13–15^ which include polysaccharides, proteins and lipids. The capacity of EPS to induce larval settlement and metamorphosis has been widely studied since 1982 when Kirchman et al. discovered that extracellular polysaccharides produced by biofilms can induce Polychaeta larval metamorphosis.^16^ Fatty acids are shown to prompt settlement and metamorphosis of tube worm *Phragmatopoma califomica* larvae.^17^ Settlement-inducing protein complex (SIPC) can induce larval settlement of the barnacle, *Balanus amphitrite.*^18^ However, this process is not well explained at the molecular level. One of the mechanisms for secretion of EPS is their release through the apparently spontaneous generation of outer membrane-derived vesicles. This is a prevalent secretion mechanism in Gram-negative bacteria, outer membrane vesicle (OMV).^19^ In this process cellular substances (nucleic macromolecules acids, enzymes, lipopolysaccharides, and phospholipids) are released into the extracellular space through the formation and release of outer membrane vesicles (OMVs), forming parts of biofilms, and play a role in larval attachment and metamorphosis of marine invertebrates.^20^

OMVs are structures consisting of a surface composed of lipopolysaccharides (LPS) and a phospholipid bilayer,^21^ enclosing nucleic acids,^22^ proteins,^23^ quorum sensing (QS) signaling molecules,^24^ and other components.^25,26^ Bacteria transmit substances and information to other prokaryotes and eukaryotes through the OMVs^27–29^ and it plays an important role in communication with neighboring bacteria and the environment.^30,31^ Using OMVs as a delivery mechanism provides several benefits over simple secretion. They can be precisely directed to specific destinations using receptors and maintain cargo integrity during long-distance travel shielding from environmental stress.^32^ Given the suitability of OMVs for intercellular delivery and its ubiquity in bacteria, it has gained attention for its interactions between bacteria and invertebrate larvae. In 2017, Freckelton et al. discovered the presence of extracellular vesicles in the inducing components of three bacterial strains affecting settlement in the serpulid polychaete *Hydroides elegans*, and suggested that bacterial extracellular vesicles play a crucial role in inducing settlement and metamorphosis of benthic marine larvae.^33^ Recently, it was determined that LPS in OMVs plays a crucial role in *H. elegans* larval metamorphosis through enzyme treatment and analysis of cell envelope components.^34^ Our previous study revealed that the VgrG protein in the T6SS of *Leisingera aquaemixtae* promotes the settlement of mussel *M. coruscus* by recruiting OMVs.^35^ However, the specific role that OMVs play in biofilm regulation of larval metamorphosis and the mechanisms that regulate OMVs remain unclear.

As a unique second messenger in bacteria, c-di-GMP relays cell-cell signals and environmental cues, regulating bacterial motility, biofilm formation, and secretion of EPS.^36^ C-di-GMP is biosynthesized by diguanylate-cyclase featuring a conserved GGDEF domain and its degradation is facilitated by phosphodiesterases that possess an EAL domain.^37^ Typically, these two structural domains with opposite activities are found together, exhibiting a predominant activity.^38^ In the genome of *P. marina*, we screened a gene containing GGDEF and EAL domains, named *cdgB*. Previous studies have found a significant correlation between c-di-GMP levels of *P. marina* and metamorphosis rate of mussel larvae. The bacterial gene *01912* associated with polysaccharide biosynthesis in *P. marina* modulates mussel settlement by regulating colanic acid production through c-di-GMP moderation.^39^ The expression of the cellulose synthesis gene *bcsQ* in *P. marina* could affect c-di-GMP concentration, governing exopolysaccharide secretion and biofilm formation, and regulates mussel settlement indirectly.^40^ *P. marina* strain deficient in the OMV synthesis gene *tolB* exhibited decreased c-di-GMP content and reduced biofilm-inducing activity for mussel plantigrade settlement.^41^ However, there is a lack of studies on the direct effect of the bacterial c-di-GMP genes on the settlement and metamorphosis of mussel larvae, and the molecular mechanism regarding the specific relationship between c-di-GMP, OMVs, and mussel settlement and metamorphosis remains unknown.

**Fig. 1:**
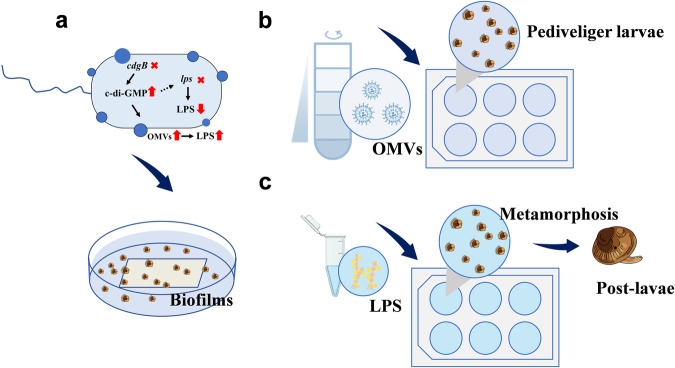
Study design for the partial experiment. **a** A model for the regulation of the bacterial c-di-GMP related gene *cdgB* on OMVs and LPS and biofilm experiments. **b** OMV exposure experiments **c** LPS exposure experiments.

*P. marina* ECSMB14103 is a strain widely distributed in the marine environment, and exhibits stable inducible activity concerning mussel larval settlement and metamorphosis.^42^ Therefore, we used *P. marina* ECSMB14103 as a model bacterium and aim to elucidate whether the bacterial biofilm-induced larval metamorphosis is governed by c-di-GMP related gene, *cdgB*, occurs through OMVs. We investigated the effects of the *cdgB* gene on bacterial biofilm properties and composition, particularly the changes in EPS and OMV contents. Then, we tested the consequences of the deletion of *cdgB* on biofilm-induced activity. The role of OMVs in inducing larval metamorphosis was verified by isolation and extraction of OMVs. Considering the critical role of LPS in OMVs, we conducted bioassays to clarify the LPS synthesis gene *lps* and LPS responsible for the altered inducing activity (Fig. 1). Addressing these questions will help to provide new insights into the molecular mechanisms by which bacterial biofilms regulate larval settlement and metamorphosis.

## Results

### Deletion of the *cdgB* gene results in elevated c-di-GMP level, decreased bacterial motility, and enhanced biofilm formation ability

**Fig. 2:**
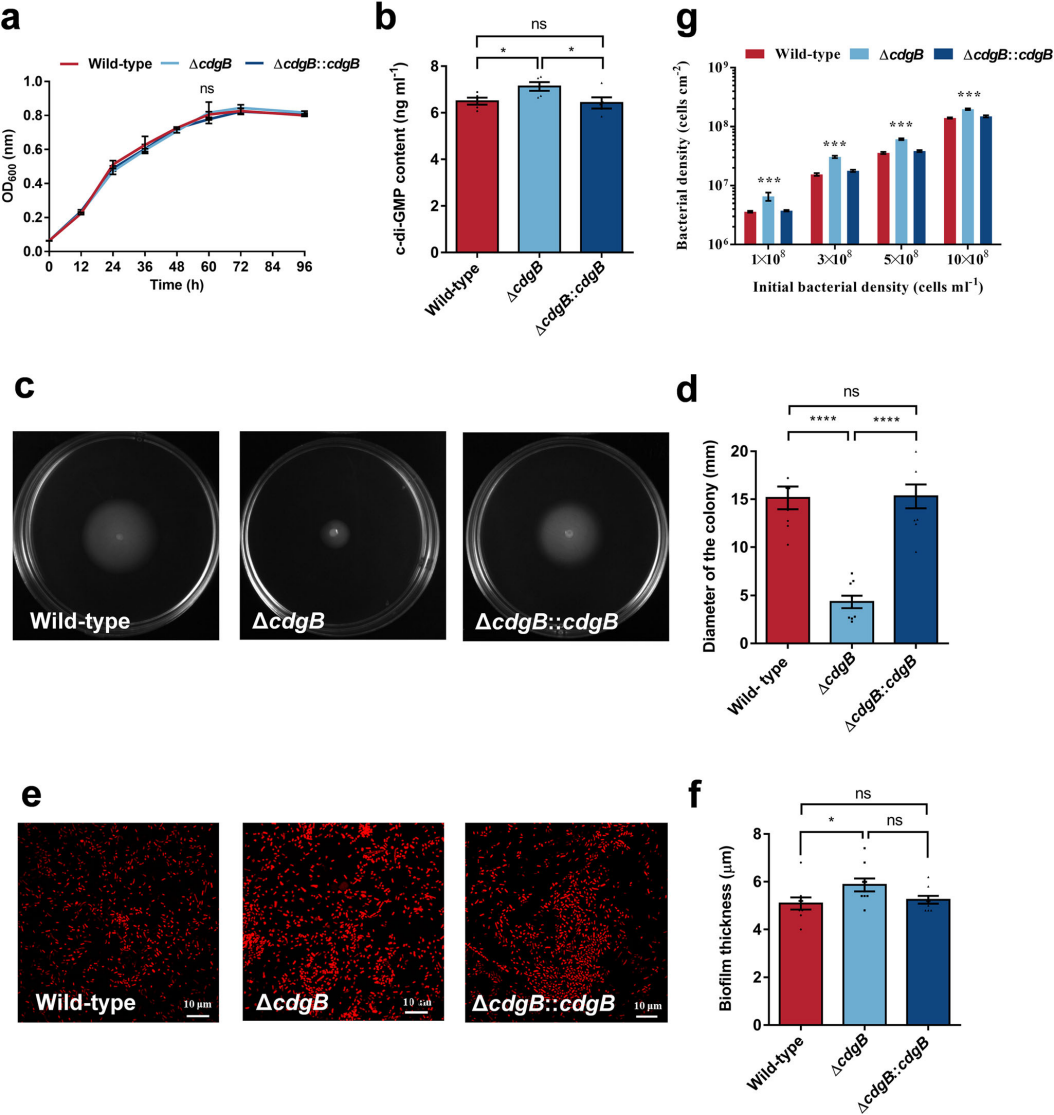
Changes in bacterial characteristics after deletion of the *cdgB* gene. **a** Consistent bacterial growth ability (*n* = 3). **b** Increased c-di-GMP concentration in Δ*cdgB* (*n* = 5). **c**, **d** Reduced swimming motility of Δ*cdgB*, **c** Image of bacterial motility. **d** Quantification of bacterial swimming motility (*n* = 9) **e**, **f** Enhanced biofilm formation ability of Δ*cdgB*, (**e**) propidium iodide staining image of biofilm formation. Scale bars indicate 10 μm. **f** Quantification of biofilm formation (*n* = 9). **g** bacterial density of biofilm at different initial density. Data were expressed as the means ± SEM. Statistical differences were determined separately by Wilcoxon test for each pair (**a**, **f**) and Student’s *t*-test (**b**, **c**). *p < 0.05, ***p < 0.001, ****p < 0.0001, and ns means not significant (*p* > 0.05).

The *cdgB* gene deletion and complementary strains were successfully constructed (Supplementary Fig. 1a, b). All three strains exhibited comparable growth capabilities (Fig. 2a). However, the strain with the *cdgB* gene deletion exhibited a notable elevation in c-di-GMP levels, with an approximately 17.50% elevation (Fig. 2b). Upon complementation of the *cdgB* gene, the c-di-GMP contents were restored to the levels observed in the wild-type strain.

The deletion of the *cdgB* gene resulted in a remarkable reduction in bacterial motility (Fig. 2c, d) and an increase in biofilm thickness (Fig. 2e, f) and bacterial density (Fig. 2g), indicating enhanced biofilm formation ability. Statistical analysis revealed that the Δ*cdgB* strain exhibited a 71.45% decrease in motility, as represented by the diameter of the bacterial swarming zone, while the complementary *cdgB* strain did not show a significant difference (Fig. 2d). The biofilm formation capacity was assessed using the following parameters: the bacterial density of the biofilm after formation at various initial concentrations and the thickness of the biofilm. The bacterial density in the Δ*cdgB* biofilm was significantly higher than that of the wild-type and complementary strains at all four concentrations. The biofilm thickness of the Δ*cdgB* strain was approximately 5.87 ± 0.27 μm, representing a 15.28% increase over the wild-type (Fig. 2f). The complemented strain exhibited a slightly lower biofilm thickness compared to Δ*cdgB*, but no significant difference (Fig. 2f).

### The Δ*cdgB* strain exerts higher EPS and OMV secretion ability

**Fig. 3:**
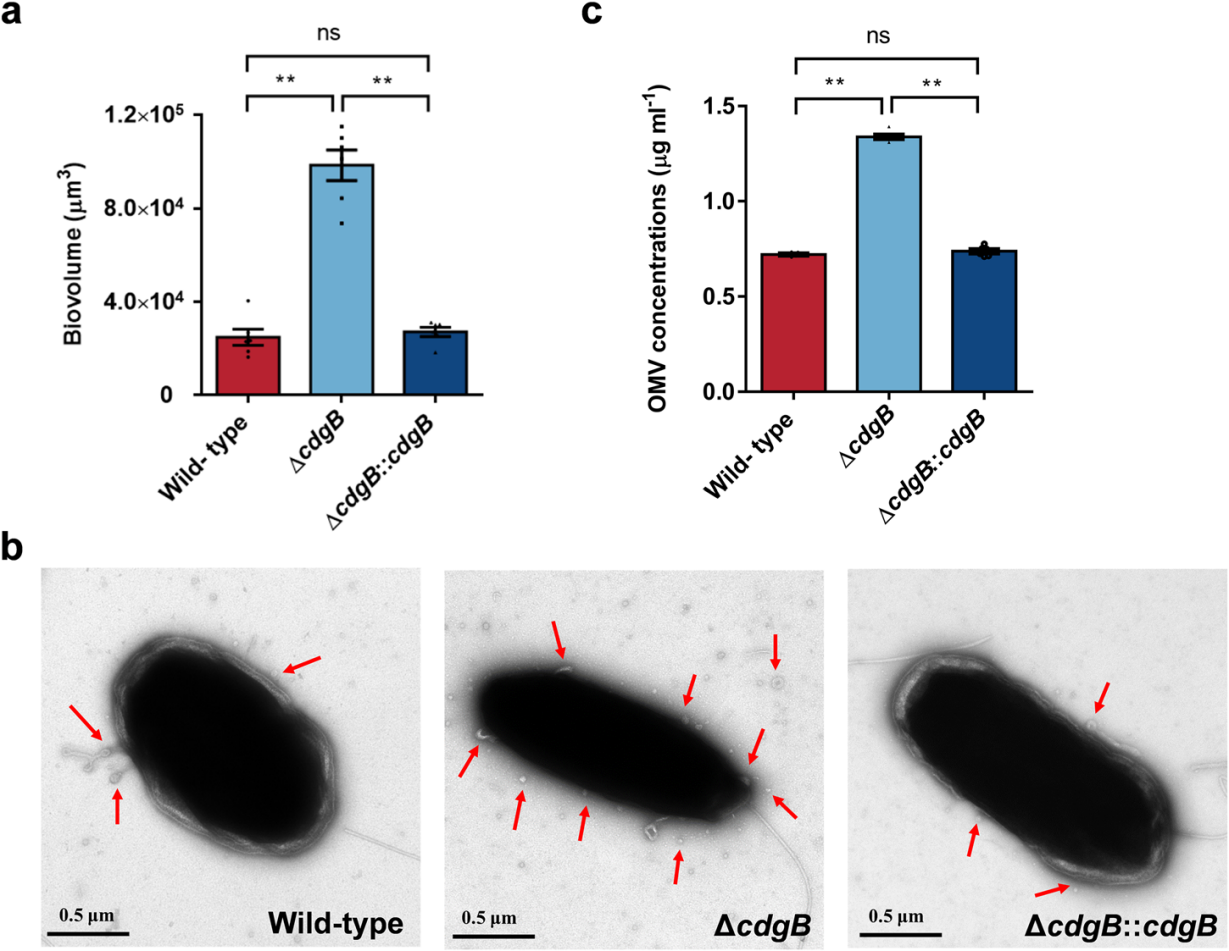
Changes in bacterial EPS content due to *cdgB* deletion. **a** Increased major component content of EPS (polysaccharides, proteins and lipids) in Δ*cdgB* biofilm (*n* = 6), **b**, **c** Elevated content of OMVs in Δ*cdgB*, (**b**) TEM images of three bacterial cells obtained from biofilms. The Δ*cdgB* cells exhibit an increased presence of spherical membrane structures in their surroundings. Scale bars indicate 0.5 μm. **c** OMV quantification of three bacterial strains (*n* = 6). Data were expressed as the means ± SEM. Statistical differences were determined by Wilcoxon test for each pair. **p < 0.01 and ns means not significant (*p* > 0.05).

In this study, the content of EPS in bacterial biofilms was represented by the sum of α-polysaccharides, β-polysaccharides, proteins, and lipids. Quantification of the EPS on the bacterial biofilm revealed that the extracellular product content of Δ*cdgB* was significantly elevated by approximately 2.96-fold (Fig. 3a), and the EPS content recovered to that of the wild-type after *cdgB* gene complementation. Morphological changes of the bacteria were observed through transmission electron microscopy (TEM). Compared to wild-type and complementary strains, significantly increased numbers of spherical vesicle structures (OMVs) in Δ*cdgB* were observed on and around the cell surface (Fig. 3b). Consequently, quantification of OMV content on the bacterial biofilms revealed that the wild-type, Δ*cdgB*, and Δ*cdgB*::*cdgB* strains had OMV contents of approximately 0.72, 1.34, and 0.74 μg ml^−^^1^, respectively (Fig. 3c). The deletion of *cdgB* gene resulted in an 85.65% increase in OMV content, while the complementary and wild-type strain showed no notable difference in OMV content (Fig. 3c). Correlation analysis between the c-di-GMP and OMV contents on the three strains revealed a highly significant correlation between the bacterial c-di-GMP levels and the OMV content on the biofilm (*r* = 0.7335, *p* = 0.0019, Supplementary Table 1).

### Deletion of the *cdgB* gene enhances the ability of biofilms to induce larval attachment and metamorphosis

**Fig. 4:**
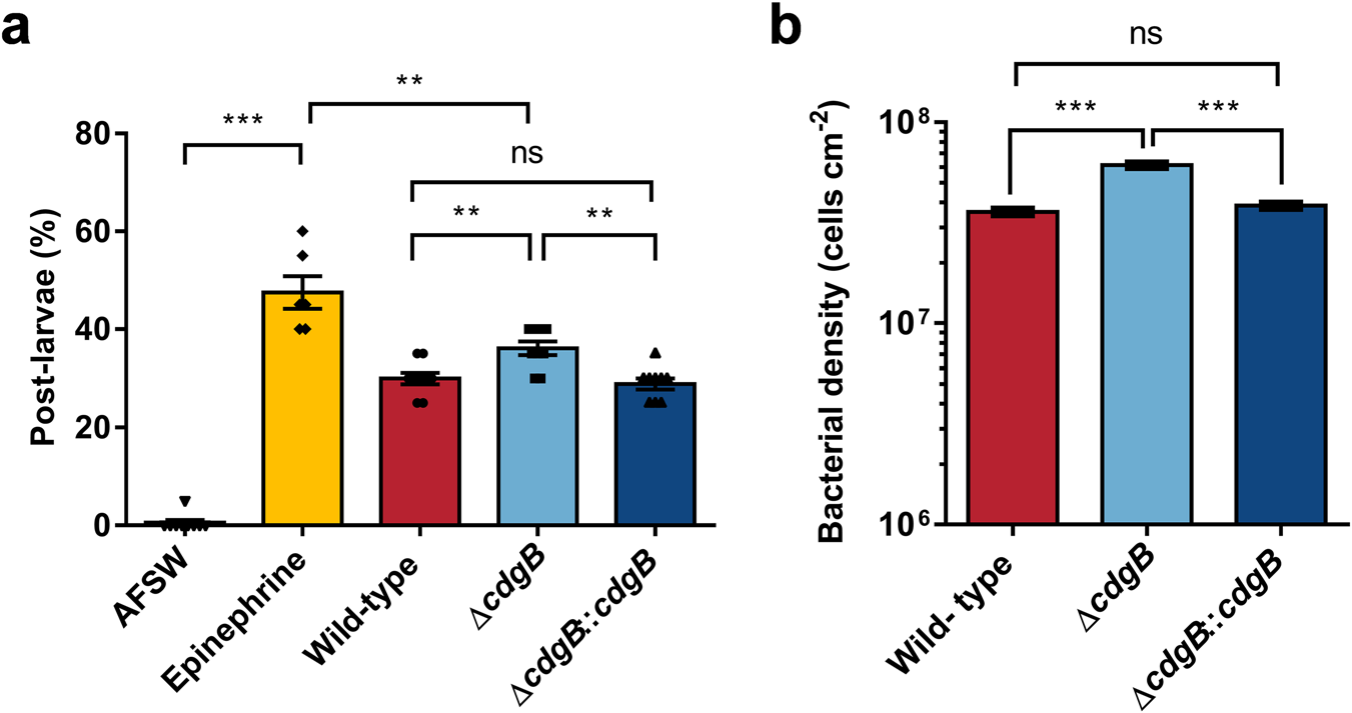
Deletion of the *cdgB* gene alters the inductive effect of bacterial biofilm on larval metamorphosis rate. **a** Increased inductive activity in Δ*cdgB*, with complementary bacteria showing similar inductive activity to wild-type bacteria (the group of Epinephrine: *n* = 6; other groups: *n* = 9). AFSW: Autoclaved filtered seawater, Epinephrine: Solution of Epinephrine at a test concentration of 10^−^^4^ mol l^−^^1^, the same applies to the following. **b** At an initial density of 5 × 10^8^ cells cm^−^^2^, the bacterial density of Δ*cdgB* after 48 h of biofilm formation is higher compared to wild-type and complementary bacteria (*n* = 30). Data were expressed as the means ± SEM. Statistical differences were determined by Wilcoxon test for each pair. **p < 0.01, ***p < 0.001, and ns means not significant (*p* > 0.05).

Epinephrine induction experiments indicated that the larvae were capable of undergoing metamorphosis. After 72 h of epinephrine induction, the metamorphosis rate of the larvae reached 47.50% (Fig. 4a). The Δ*cdgB* biofilms showed a 20.37% increase in induction rate than that of the wild-type biofilms (30.00%). In contrast, the Δ*cdgB*::*cdgB* biofilm exhibited no significant change (Fig. 4a). There was a notable correlation between OMV content and larval metamorphosis rate (*r* = 0.7295, *p* = 0.0006, Supplementary Table 1). Additionally, the final bacterial density of the Δ*cdgB* biofilm was higher than that of the wild-type and Δ*cdgB*::*cdgB* (Fig. 4b).

### OMVs in EPS induce larval metamorphosis of *M. coruscus*

**Fig. 5:**
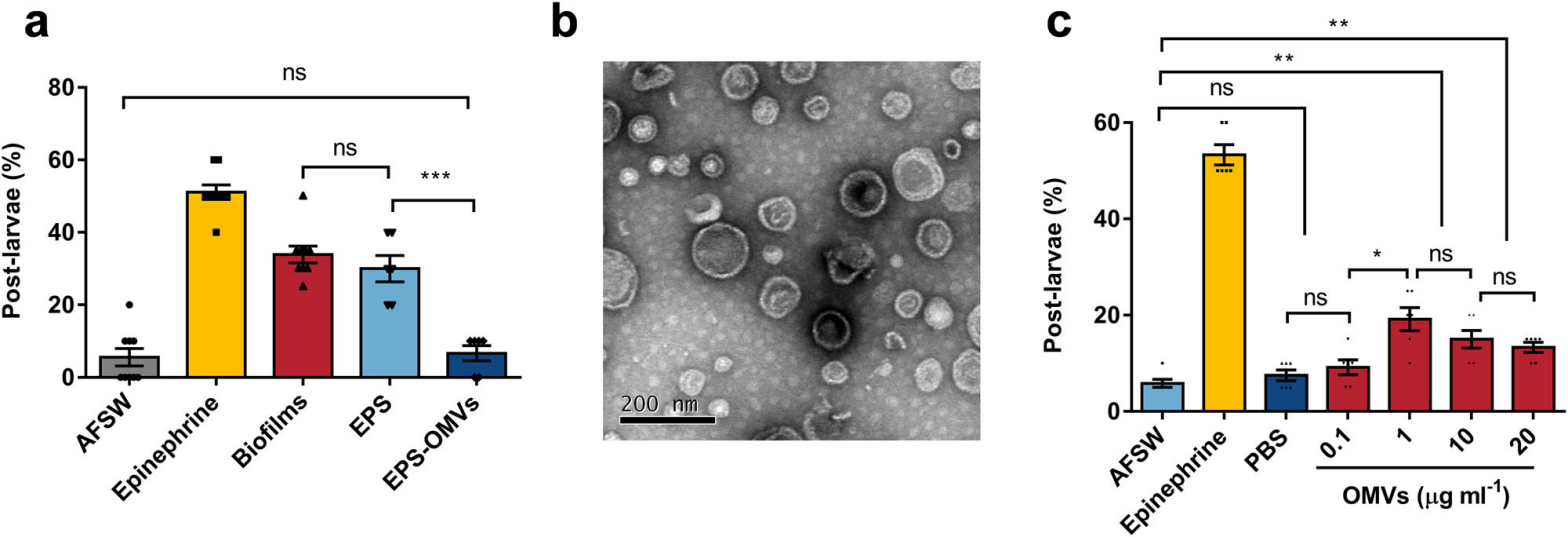
Inductive effect of OMVs on larval metamorphosis. **a** No difference in inducing effect between EPS and biofilms. EPS - OMVs has no significant impact on larval metamorphosis compared to AFSW, indicating no inducing effect (the group of AFSW, Epinephrine, and Biofilms: *n* = 9; the group of EPS and EPS-OMVs: *n* = 6). EPS-OMVs: EPS removal of OMVs. **b** TEM image of purified OMVs extracted from *P. marina*. Scale bar indicates 200 nm. **c** PBS: PBS solution used for OMV dissolution, showing no significant difference from the AFSW group. Inductive effects observed for OMVs at concentrations of 1, 10, and 20 μg ml^−^^1^ (*n* = 6). Data were expressed as the means ± SEM. Statistical differences were determined by Wilcoxon test for each pair. *p < 0.05, **p < 0.01, ***p < 0.001, and ns means not significant (*p* > 0.05).

To verify the relationship between OMVs and larval metamorphosis, we extracted the EPS of wild-type strains, removed the OMVs in the EPS by ultracentrifugation to obtain the EPS without OMVs, and stimulated the larvae with EPS and EPS without OMVs. It was found that EPS containing OMVs was able to induce larval metamorphosis with a metamorphosis rate of 30.00%, which was not significant statistically different from the induction activity of the biofilms of the wild-type strain (33.89%) (Fig. 5a). Remarkably, what was most surprising was that the induction activity of the EPS without OMV was only 6.67%, which was similar to the autoclaved filtered seawater (AFSW) control group (5.56%) without significant differences (Fig. 5a).

To further confirm the inducing activity of OMVs, OMVs were extracted and purified from the biofilms of the wild strains of *P. marina*. Subsequently, the structural integrity of these vesicles was assessed using TEM. The extracted and purified OMVs retained their intact spherical structure. (Fig. 5b). Following this, the larvae were directly stimulated with the four concentrations (0.1, 1, 10, and 20 μg ml^−^^1^) of purified OMVs. When larvae were exposed to AFSW containing OMVs extracted from the wild-type strain of *P. marina*, some larvae transitioned from swimming to a stationary or crawling state. Velum were shed, juvenile/adult shell dissoconch growth occurred, and gills gradually formed, which are counted as post-larvae^9^ (supplementary Fig. 2). At high concentration of 20 μg ml^−^^1^, the larval survival rate at 72 h was 87% (Supplementary Fig. 3a). The highest metamorphosis rate was observed at 72 h, with the best induction activity observed at an OMV concentration of 1 μg ml^−^^1^, resulting in approximately 19.17% metamorphosed larvae (Fig. 5c). At an OMV concentration of 0.1 μg ml^−^^1^, the larval metamorphosis rate showed no significant difference compared to the negative control groups (AFSW and PBS), indicating no induction activity of OMVs at this concentration (Fig. 5c). Exposure to OMVs at concentrations of 10 and 20 μg ml^−^^1^ resulted in metamorphosis rates of 15.00% and 13.33%, respectively (Fig. 5c).

### LPS induces larval metamorphosis

**Fig. 6:**
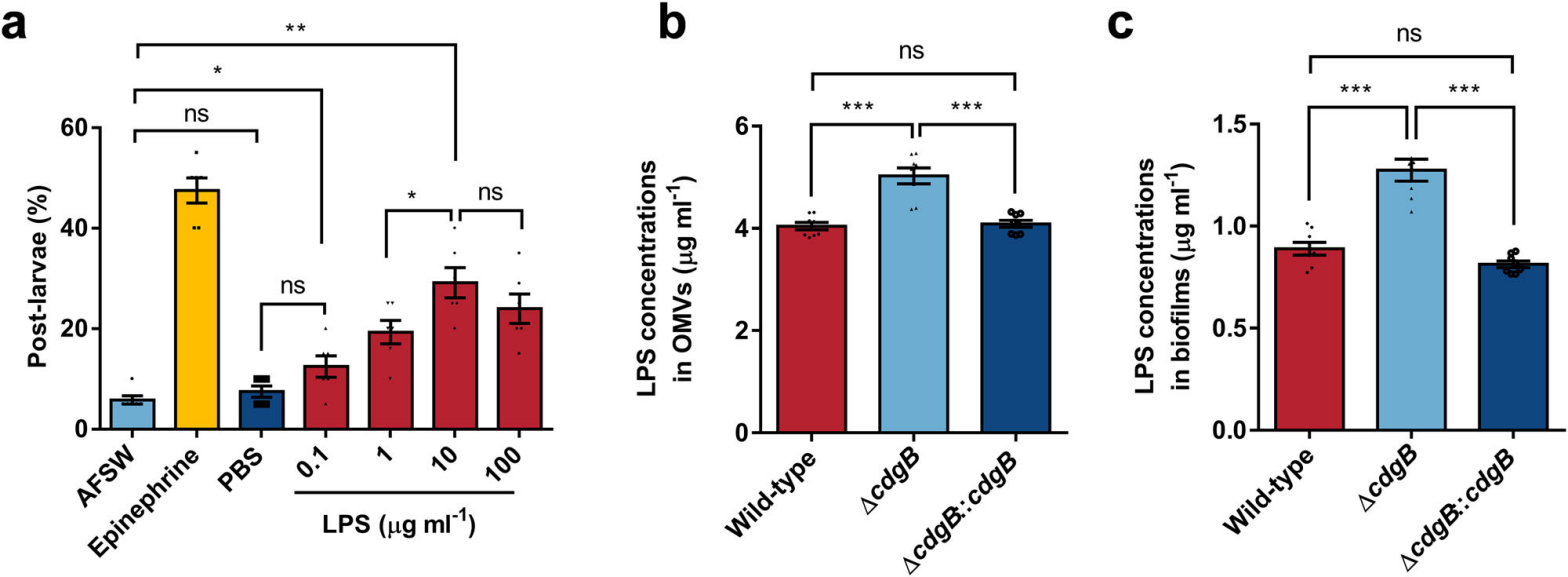
Inductive impact of LPS on larval metamorphosis. **a** Inductive effect of LPS independently on larvae (*n* = 6). **b** The LPS concentration in 1 ml of OMVs at a concentration of 1000 μg ml^−^^1^ (*n* = 8). **c** Content of LPS in biofilms, grouped in sets of twelve biofilm slides (*n* = 8). Data were expressed as the means ± SEM. Statistical differences were determined by Wilcoxon test for each pair. *p < 0.05, **p < 0.01, ***p < 0.001, and ns means not significant (*p* > 0.05).

Since the extracted OMVs were found to be structurally intact and exhibited inducible activity, we formulated a hypothesis suggesting that the active agents responsible for induction within OMVs are situated within the LPS and phospholipid bilayer on the external surface of these vesicles. Building upon Freckelton’s prior research,^34^ it is conceivable that LPS serves as an effector of OMVs, facilitating metamorphosis activation. To verify this conjecture, our initial step involved the isolation of LPS from *P. marina*. When larvae were exposed to LPS alone, they exhibited a dose-dependent effect, with the metamorphosis rate increasing with increasing LPS concentration. The highest metamorphosis rate was observed at 10 μg ml^−^^1^, reaching 29.00% (Fig. 6a). However, at 100 μg ml^−^^1^ concentration, there was a reduction in the metamorphosis and survival rate, the latter decreased to 60.83% (Supplementary Fig. 3b).

We then aimed to investigate whether LPS was also regulated by gene *cdgB*. Initially, we assessed the LPS content within the biofilms and OMVs of the three strains, wild-type, Δ*cdgB*, and complementation of *cdgB*. The results indicated an increase in LPS content by 24.34% in the OMVs (Fig. 6b) and by 43.30% in the biofilms (Fig. 6c). No significant distinction existed between the complementary and the wild-type strains (Fig. 6b, c). Correlation analyses were conducted between the level of c-di-GMP and LPS. The levels of c-di-GMP showed significant correlations, both in terms of the LPS content on the biofilm’s surface (*r* = 0.6357, *p* = 0.0109) and within the LPS content of OMVs (*r* = 0.6130, *p* = 0.0151) (Supplementary Table 2). Remarkably, it also exhibited a significant correlation between the inducing activity of biofilm and LPS content in OMVs (*r* = 0.5643, *p* = 0.0041, Supplementary Table 3).

### Deletion of the *lps* gene exhibits distinct morphology, elevated c-di-GMP level, reduced bacterial motility, and an enhanced ability for biofilm formation

**Fig. 7:**
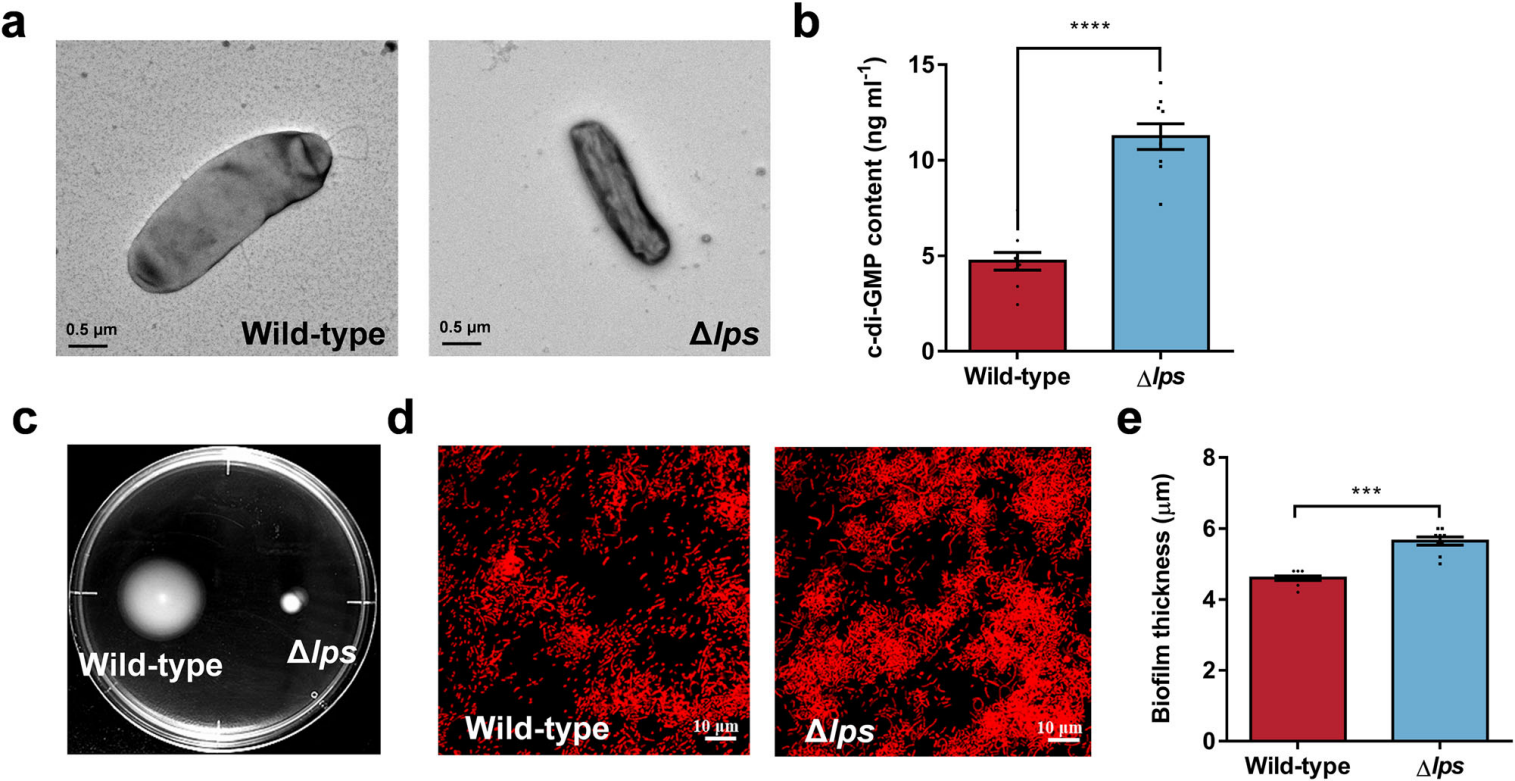
Changes in biological characteristics of LPS gene-deficient strain. **a** TEM images of wild-type and Δ*lps* strains. Scale bars indicate 0.5 μm. **b** C-di-GMP contents in biofilms of two strains (*n* = 9). The statistical differences were determined by Student’s *t*-test. **c** Bacterial swimming motility indicated by the diameter of the growth zone on motility medium. **d** Propidium iodide staining biofilm image of wild-type and Δ*lps*. **e** Biofilm thickness (*n* = 9) of wild-type and Δ*lps* strains. Data were expressed as the means ± SEM. Statistical differences were assessed using the Wilcoxon test for each pair. ***p < 0.001 and ****p < 0.0001.

To explore the molecular mechanism of the *lps* gene and comprehend the underlying mechanism of LPS formation, we constructed the Δ*lps* strain through gene-knockout technology (Supplementary Fig. 1c). Distinct morphological differences were noted between the two strains. The wild-type strains exhibited a fuller and smoother shape, appearing larger in size (Fig. 7a). In contrast, the Δ*lps* strains displayed an elongated and shriveled body morphology (Fig. 7a). Moreover, the c-di-GMP level was elevated by approximately 1.38-fold (Fig. 7b). Simultaneously, bacterial motility was reduced (Fig. 7c), while biofilm formation was enhanced (Fig. 7d, e).

### The Δ*lps* strain decreases the ability of biofilms to induce larval settlement and metamorphosis

**Fig. 8:**
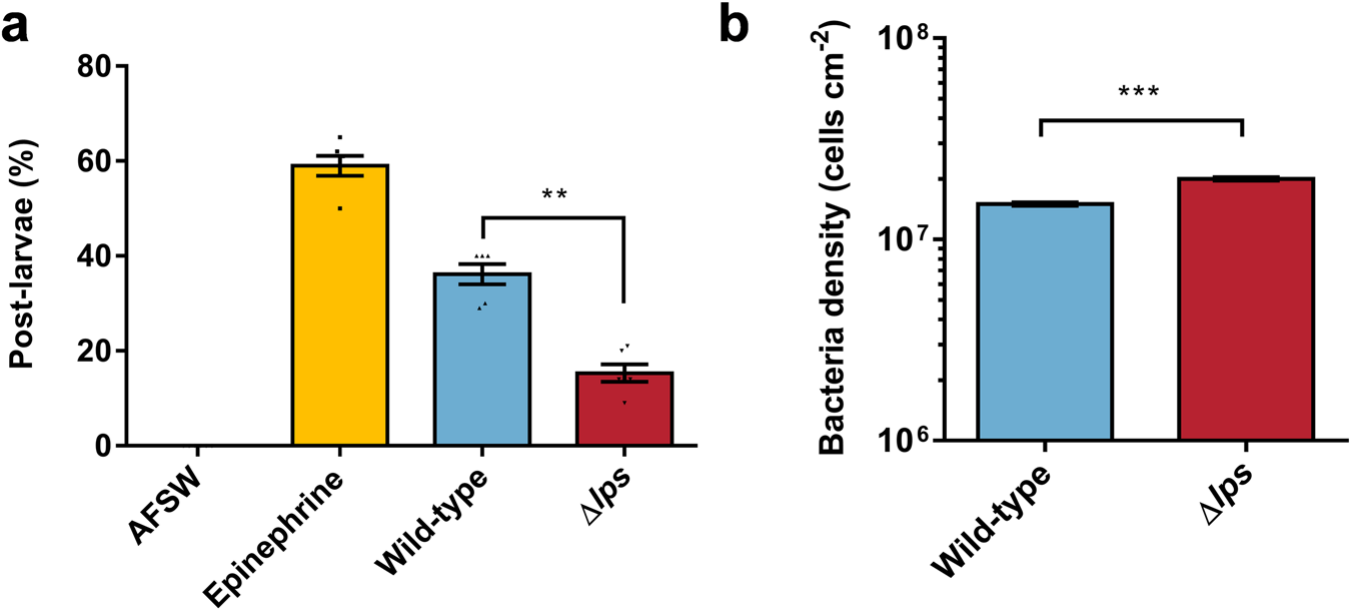
Deletion of the *lps* gene alters the inductive effect of bacterial biofilm on larval metamorphosis rate. **a** Increased inductive activity of Δ*lps* biofilm (*n* = 6). **b** At an initial density of 5 × 10^8^ cells cm^−^^2^, the bacterial density of Δ*lps* after 48 h of biofilm formation is higher compared to wild-type bacteria (*n* = 30). Data were expressed as the means ± SEM. Statistical differences were determined by Wilcoxon test for each pair. **p < 0.01 and ***p < 0.001.

Subsequent evaluation of larval attachment metamorphosis induced by bacterial biofilms revealed that the Δ*lps* biofilm exhibited an inducing ability of only 15.33%, marking a reduction of approximately 57.60% to the wild-type biofilm (Fig. 8a). Conversely, the final bacterial density within the biofilm, initiated at a concentration of 5 × 10^8^ cells ml^−^^1^, exhibited an increase (Fig. 8b).

### Reduction in LPS content leads to decreased inducing activity of Δ*lps* biofilms

**Fig. 9:**
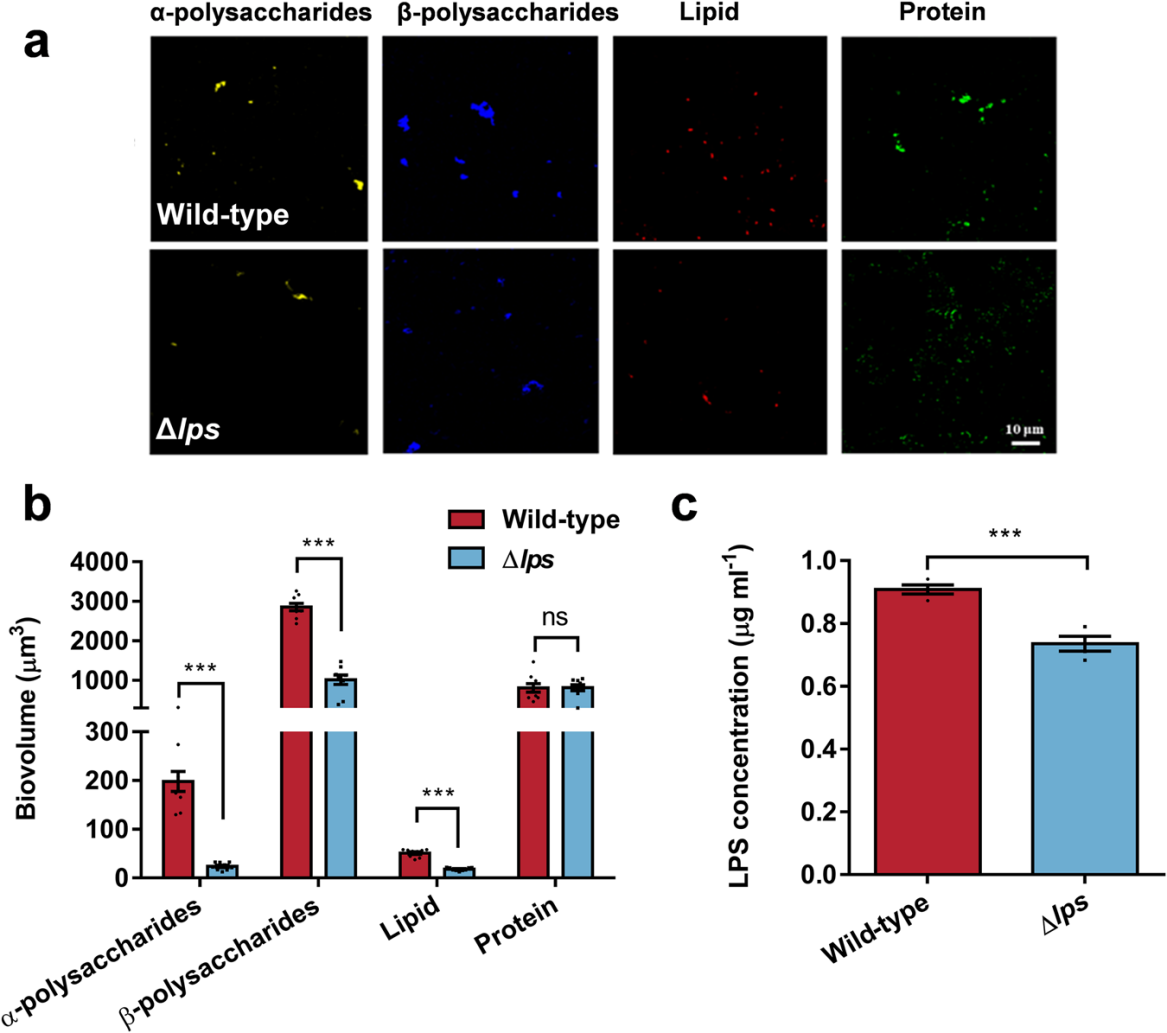
Changes in Extracellular polymeric substance content due to *lps* gene deletion. **a** Distribution of α-polysaccharides, β-polysaccharides, lipids, and proteins on the biofilm of wild-type and Δ*lps* strains, (**b**) along with biomass analysis (*n* = 9). Scale bar indicates 10 μm. Statistical differences were determined by Wilcoxon test. **c** The extracellular LPS content in both strains (*n* = 4). Data were expressed as the means ± SEM. Statistical differences were discerned using the Student’s t-test. ***p < 0.001 and ns means not significant (*p* > 0.05).

Firstly, confocal laser scanning microscope (CLSM) analysis of the biofilms of the strain revealed a decrease in the content of extracellular polysaccharides and lipids in the Δ*lps* (Fig. 9a, b). This change was hypothesized to be attributed to the reduction in LPS content. Quantification of the LPS in biofilm demonstrated a decrease of 19.01% following the deletion of the *lps* gene (Fig. 9c). Correlation analysis indicated a significant correlation between the inducing activity of the biofilm and the LPS content (*r* = 0.7545, *p* = 0.0305, Supplementary Table 4).

## Discussion

In current experiment, we clarified the molecular mechanisms of c-di-GMP regulation of biofilm-induced larval settlement and metamorphosis. We directly demonstrated that deletion of *cdgB* gene in *P. marina* strain could alter the level of c-di-GMP, affecting secretion of EPS, especially the production of OMVs. Additionally, OMVs were found to be the main cause of EPS-induced larval metamorphosis, and LPS was the main active component of OMVs-induced metamorphosis in mussels. Moreover, we studied the synthesis mechanism of LPS and found that the content of LPS was affected by c-di-GMP, and directly regulated by *lps* gene. The absence of *lps* led to a decrease in LPS content and consequently reduced the biofilm activity of inducing larval settlement and metamorphosis.

Since Zobell et al. first discovered the important role of biofilm in larval settlement in 1935,^43^ after decades of research on the interactions between biofilms and invertebrates, we still know little about the molecular mechanisms on biofilms regulate larval settlement and metamorphosis. Huang and Hadfield found that small differences between different strains of the same bacterium were responsible for inducing larval metamorphosis.^44^ Therefore, investigation of bacterial genes involved in controlling larval settlement and metamorphosis comes into focus. A set of genes encoding the bacterial phage tail-like structures identified in the bacterium *Pseudoalteromonas luteoviolacea*, metamorphosis-associated contractile structure^45^ genes, responsible for regulating metamorphosis in tubeworms.^46^ However, only very few bacteria have this structure,^33^ which contradicts the fact that biofilms, which are ubiquitous in the oceans, commonly induce larval metamorphosis. Thus, we wanted to know the universal mechanisms by which biofilms induce metamorphosis in larvae.

Biofilm formation results from large numbers of interacting sensory signals — for example, QS, chemotaxis, and also second messenger signaling system.^47,48^ In *Vibrio cholerae*, QS controlled biofilm formation through the modulation of c-di-GMP contents.^49^ In *Pseudomonas aeruginosa*, the expression of QS regulated genes was elevated when intracellular c-di-GMP levels are reduced.^50^ Previous researches demonstrated that QS signals produced by bacteria could control larval settlement.^51,52^ However, there is a lack of evidence to elucidate the relationship between c-di-GMP, QS signals and larval settlement.

C-di-GMP is a ubiquitous bacterial signaling molecule.^53^ In our previous studies, it was observed that deletion of polysaccharide genes resulted in consistent and correlated changes in c-di-GMP levels and the ability of biofilms to induce larval settlement and metamorphosis.^39,40^ However, these studies did not elucidate the specific mechanisms by which these polysaccharide-related genes regulate c-di-GMP levels, and there was still no direct evidence demonstrating the exact role of c-di-GMP related genes in the process of larval settlement and metamorphosis. The *cdgB* gene encodes an enzyme responsible for the c-di-GMP content in *P. marina*, and it was presumed to be responsible for c-di-GMP degradation. The protein function of *cdgB* gene of *P. marina* was identified as diguanylate phosphodiesterase, similar to the role of PA1727 in *Pseudomonas aeruginosa* PAO1 bacterium. PA1727, also known as *mucR*, is associated with intracellular c-di-GMP content and host-interaction.^54,55^ In previous studies, MucR1, identified as a global pleiotropic regulator, played a crucial role in the *Sinorhizobium*-Soybean Symbiosis.^54^ The MucR1 transcriptionally repressed diguanylate cyclase, leading to a reduction in c-di-GMP levels and facilitating the prokaryote-eukaryote interactions.^54^ Consequently, we further investigated the gene function of *cdgB*. After deletion of the *cdgB* gene, the bacterial motility decreased, and the ability to form biofilms was enhanced, which is consistent with previous studies.^56,57^ Upon complementation of the *cdgB* gene, c-di-GMP levels were restored, accompanied by the recovery of other bacterial characteristics. This also indicates that the changes in bacterial motility and biofilm formation ability were caused by the absence of the *cdgB* gene. There is increasing evidence supporting the crucial role of c-di-GMP in bacterial-host interactions.^39–41,58^ In nematode *Caenorhabditis elegans*, c-di-GMP serves as a key factor regulating the transition of bacteria from a free-living state to a host-associated state.^58^

Given the increased c-di-GMP content and enhanced biofilm-forming capability of Δ*cdgB*, we postulated that there was an augmentation in the secretion of EPS, a major component of biofilms.^59^ Our supposition was substantiated by the determined EPS quantification results (Fig. 3a). It is worth noting that when we performed TEM observations on the morphology of the bacteria, we observed a notable increase number of OMV structures surrounding the mutant strain Δ*cdgB*. Therefore, we analyzed the OMV content in the biofilm of mutant strain and found an increase in the production of OMVs, showing a highly significant correlation between OMVs and c-di-GMP levels. We demonstrated that the deletion of genes involved in c-di-GMP degradation leads to an increase in OMV production. A positive correlation between c-di-GMP and OMV content has been observed in a non-PQS producing species (*Pseudomonas* quinolone signal, PQS is a mechanism controlling OMV generation in *P. aeruginosa.*^60^) However, reports indicated that c-di-GMP inhibits OMV production in *P. aeruginosa* and *E. coli.*^61^ In *P. aeruginosa*, a decrease in c-di-GMP levels leads to a significant increase in extracellular PQS and OMV production. On the other hand, in non-PQS producing *E. coli*, c-di-GMP may affect the production of a membrane perturbing small molecule, thereby altering the levels of OMVs, so it has been suggested that c-di-GMP exerts a conserved negative regulation on OMV production.^61^ In contrast, our findings suggest that in non-PQS producing strain, *P. marina*, c-di-GMP does not negatively but positively regulate OMV production.

We assessed the induction activity of mutant bacterial biofilms. Biofilms with elevated c-di-GMP levels exhibited significantly higher induction capacity. High c-di-GMP levels stimulate the expression of EPS.^53^ Whereas, extensive researches have underscored the pivotal role of EPS in attachment metamorphosis within invertebrates.^62–64^ Therefore, a pertinent question arises: does the biofilm induction activity pivot on the EPS? To elucidate this, we tested the inducible activities of two types of EPS, one extracted from a wild-type strain and the other extracted and then subjected to ultra-high-speed centrifugation to remove OMVs and retained other polymeric substances including polysaccharides, proteins, and etc (EPS-OMVs). The results were interesting in that the induction effect of EPS was roughly equivalent to that of the entire biofilm, whereas the inducing effect of EPS-OMVs decreased by 77.78% (Fig. 5a). Although analogous studies have previously demonstrated the inducible potential of OMVs in larval metamorphosis.^33–35,41^ It has been observed that OMVs can induce settlement of *H. elegans* and *M. coruscus*, and speculating the mechanism of OMV-mediated larval metamorphosis is broadly common.^33^ However, OMVs were proposed as the principal contributors to the inducing effects facilitated by bacterial EPS in this study. Subsequently, our efforts involving OMVs isolation, purification, and induction experiments reaffirmed the inducibility of OMVs in larval metamorphosis independently. The optimal concentrations of OMVs and LPS to induce *M. coruscus* larval metamorphosis were determined as 1 μg ml^−^^1^ (Fig. 5c) and 10 μg ml^−^^1^ (Fig. 6a), respectively. However, higher concentrations resulted in a downregulation of *M. coruscus* metamorphosis rate (Figs. 5c, 6a). In the tubeworm, it was also observed that LPS of lower concentration (5 μg ml^−^^1^) exhibited more effective induction in *H. elegans* larval metamorphosis compared to higher concentrations, while concentrations higher than 50 μg ml^−^^1^ showed toxicity to larvae.^34^ Consequently, larval metamorphosis might be concentration-dependent on OMVs and LPS, and high concentrations of chemical cues, like OMVs and LPS, might exert toxicity on marine invertebrates. Further bioassay is needed.

The elevated c-di-GMP contents in bacterial biofilms led to an 20.37% increase in the induction activity on mussel larvae (Fig. 4a), while OMVs alone exhibited only 19.17% (Fig. 5c). This suggests that c-di-GMP can influence larval metamorphosis through other substances. In our previous studies, we discovered that c-di-GMP affects larval metamorphosis by modulating the production of various EPS.^39,40,65^ EPS devoid of OMVs, which, despite manifesting low inducible activity statistically indistinguishable from the control, might still harbor substances with inducible potential, such as free LPS within the EPS, as well as substances such as colanic acid and flagellin.^39,66^ In present study, EPS-OMVs exhibited markedly low inducing activity, which was inconsistent with the high inducing activity observed with single purified substances isolated from EPS in previous studies.^65,66^ We propose that the difference was due to the interplay of the components within EPS involving synergistic effects of metamorphosis-promoting and -suppressing effects, resulting in much lower induction levels compared to the cohesive inducing activity exhibited by OMVs as an integrated entity. Besides bacterial EPS, we need further research on whether c-di-GMP itself possesses induction effects, which will be the focus of our forthcoming research endeavors.

It is speculated that the larvae perceive external signals from the entire OMV structure rather than its internal components during the induction of larval metamorphosis. The results from TEM further corroborated this hypothesis showing that purified OMVs displayed intact spherical structures without any signs of breakage. Since LPS located on the external surface of OMVs, which contains a highly variable polysaccharide region referred to as the O antigen,^67,68^ likely the first component in contact with host cells. It can be hypothesized that the LPS present on OMVs may be a structural factor influencing OMV recognition.^69^ Bacterial lipopolysaccharide of *C. lytica* induced settlement and metamorphosis in *H. elegans.*^34^ The potential of LPS to induce settlement of juvenile mussels has also been tentatively demonstrated in *M. coruscus*, and the ability of Phospholipase C-treated OMVs to induce settlement of juvenile mussels was drastically reduced.^41^ In the present experiments, we demonstrated the induction activity of LPS on larval metamorphosis by direct exposure to LPS, and furthermore explored the molecular mechanisms by which LPS affects larval metamorphosis by gene knockout. We identified a previously unstudied gene, *orf02818*, in the genome of *P. marina* through homology. It was annotated as “lipopolysaccharide biosynthesis” for gene function. Consequently, we designated it as the *lps* gene. In the COGs database, *lps* was annotated as COG3206, indicating an “exopolysaccharide export protein/domain GumC/Wzc1”. Based on this information, we speculated that the *lps* gene is involved in the export process during LPS biosynthesis. The absence of *lps* gene may affect the secretion of LPS into the extracellular space, resulting in a decrease in LPS content. Therefore, deleting the *lps* gene resulted in a reduction in LPS content of biofilm and led to a decrease in strain induction activity by more than half, further emphasizing the pivotal role of *lps* gene in inducing larval metamorphosis. Upon analyzing other properties of Δ*lps* strain, we noted altered individual bacterial morphology. The reduction in LPS resulted in alteration of the outer membrane structure and bacterial thinning because LPS is a major constituent of outer membrane of Gram-negative bacteria.^70^ As a result, bacterial motility was also affected and biofilm thickness increased. We quantified the LPS content within the biofilms and OMVs of wild-type, *cdgB* gene deletion, and *cdgB* gene complementary strains. The results showed the same trend as that of c-di-GMP contents, *cdgB* gene deletion led to elevated LPS levels, while *cdgB* gene complementation restored LPS content. This suggests that LPS content is also regulated by the *cdgB* gene.

Bacterial c-di-GMP synthesis and degradation are regulated through cooperative actions of specific domains.^38^ The deletion of the *cdgB* gene disrupted the degradation of c-di-GMP, leading to an increase in its intracellular contents. Consequently, bacterial motility was reduced while biofilm formation capacity was enhanced. The elevated c-di-GMP contents resulted in an increased production of OMVs within the biofilm, thereby enhancing the biofilm’s ability to induce larval metamorphosis. Although EPS content significantly increased, our findings demonstrated that the primary inducible agents within biofilms are OMVs. LPS in OMV played the key role in inducing mussel metamorphosis, and it was regulated by the *lps* gene. Notably, this study uncovered the regulatory function of c-di-GMP related gene in controlling the levels of bacterial OMVs. Both OMVs and LPS play a crucial role in the interaction between bacterial biofilms and invertebrates. This research elucidates the relationship between c-di-GMP, OMVs, LPS and larval metamorphosis, providing the insights and strategies for understanding the impact of biofilms on the metamorphosis of marine invertebrates.

## Methods

### Strains and plasmids

Supplementary Table 5 displays the strains and plasmids employed in this experiment. The strain was *P. marina* ECSMB14103, a marine bacteria isolated from the natural biofilms of the East China Sea, and was cultured in Zobell 2216E liquid medium at 200 rpm at 25 °C. The plasmid *Escherichia coli* pK18mobsacB-Ery was cultured in Lysogeny broth liquid medium at 200 rpm at 37 °C with corresponding antibiotics.

### Larva breeding and metamorphosis assay

The larval development up to the pediveliger stage exhibits metamorphic capabilities. Consequently, in this experiment, pediveliger larvae were employed to assess the inductive activity of biofilms and other substances. The pediveliger larvae of mussel *M. coruscus*, obtained from Ningbo Yinzhou Sanwan Aquatic Seed Co., were used for the metamorphosis assay. The larval shell length was 273.76 ± 17.87 μm, and the shell height was 250.88 ± 17.10 μm. The larvae were temporarily reared at 18 °C and fed with *Isochrysis zhanjiangensis*. Epinephrine has been demonstrated to be effective in inducing larval metamorphosis of various marine invertebrates, including *M. coruscus,*^71–76^ so epinephrine was chosen as a positive control to assess the larval metamorphic capacity. Metamorphosis experiments were conducted once the larvae exhibited metamorphic capacity, while AFSW served as the negative control during the experiments. The larvae were not fed during the whole metamorphosis experiment. Biofilm experiments were carried out in Petri dishes (Ø 64.0 × 19.0 mm) (Fig. 9a), while OMV and LPS exposure experiments were conducted in six-well plates (Corning, America) (Fig. 9b, c) with a total system volume of 20 ml. Each well of the six-well plate contained a total system volume of 10 ml. OMV solutions dissolved in PBS were added to the wells in appropriate amounts based on the desired OMV concentration, resulting in final concentrations of 0.1, 1, 10, and 20 μg ml^−^^1^, respectively. The LPS experiment referred to the above. The experiments were conducted over a span of 0–96 h, with morphological and behavioral changes of the larvae recorded every 24 h. The metamorphosis rate at 72 h was ultimately used to characterize the inducing activity of the biofilm and compounds. Each experimental group consisted of six replicates, with 20 larvae in each replicate.

### Strain construction

In this study, homologous recombination was employed to perform gene knockout and the complementation was performed by introducing a replicative vector containing the wild-type gene. Firstly, the c-di-GMP related gene *cdgB* (*orf00631*) and LPS biosynthesis gene *lps* (*orf02818*) were identified within the genome of *P. marina* separately. The primers employed are detailed in Supplementary Table 5. Following the method described by ref.,^77^ the upstream and downstream fragments of the target gene were PCR-amplified using primers with corresponding restriction enzyme recognition sites. Subsequently, the PCR products were digested and then ligated into the empty vector pK18mobsacB-Ery, resulting in the recombinant vector. The recombinant vector was then introduced into *E. coli* DH5α competent cells for transformation and cultured to increase the concentration of the recombinant plasmid. The recombinant plasmid was extracted and transferred into *E. coli* WM3064 cells. Successful transformation was confirmed by mixing the transformed *E. coli* WM3064 strain with *P. marina* wild-type strain and plating the mixture on 1/2 SW-LB-DAP agar plates for conjugation transfer. Following incubation at 25 °C for 12–48 h, the colonies were diluted and streaked on Zobell 2216E plates containing erythromycin to select single colonies. PCR verification was performed using LF/SR and SF/LR primers. Validated strains were then streaked on 20% 2216E-sucrose medium for 2 days, and single colonies were picked for PCR validation using LF/LR and SF/SR primers. Finally, the confirmed knockout strains were subjected to a final verification using SF/SR, LF/SR, SF/LR, and LF/LR primers.

Subsequently, gene complementation was performed using the pBBR1MCS-Cm vector. First, the target gene fragment was amplified using down-F/up-R primers, and the PCR product was subsequently digested and ligated into into the empty pBBR1MCS-Cm plasmid, generating the recombinant complementation plasmid. The recombinant plasmid was transferred into *E. coli* WM3064 cells, and amplification sequencing was performed using pBBR-F/pBBR-R primers to validate the insertion. The *E. coli* WM3064 strain carrying the recombinant plasmid was then transferred to the mutant strain, and positive monoclonal bacteria were obtained by selection on chloramphenicol-resistant plates, resulting in the complemented strain Δ*cdgB*::*cdgB*.

### Determination of bacterial growth ability, motility and morphology

Three bacterial strains were cultured until they reached an optical density at 600 nm (OD600) of 0.8. Then, 50 μl of each culture was inoculated into 150 ml of Zobell 2216E liquid medium in shake flasks, following the previously described culturing conditions. The OD600 values of the bacterial cultures in the shake flasks were measured every 12 h within the 0–96 h time period. Each measurement was performed in triplicate.

Furthermore, the three bacterial strains were cultured to an OD600 of 0.8, and 1 μl from each culture was spotted onto motility plates to assay swimming motility, as referred to by Zeng et al.^77^. The plates were incubated at 25 °C, and once the bacterial colonies reached an appropriate size, photographs were taken, and the diameter of the bacterial swarm was measured. Nine biological replicates of each measurement were used for the experiment. TEM images of bacteria and OMV samples obtained from biofilms were captured using the negative staining technique.

### Cultivation of biofilms, counting of bacterial density, evaluation of biofilm formation, and quantitative analysis of EPS

Biofilms were cultivated following the method of Yang et al.^9^. The bacterial culture, grown for 12–16 h, was washed with AFSW to remove EPS. Bacterial density was measured, and based on the bacterial density, bacterial suspensions were prepared at initial densities of 1 × 10^8^, 3 × 10^8^, 5 × 10^8^, and 10 × 10^8^ cells ml^−^^1^. These suspensions were added to culture dishes (Ø 90.0 × 15.0 mm) and adjusted to a total volume of 20 ml with AFSW. The biofilms were then cultivated at 18 °C. In each dish, three sterilized glass slides (12.7 × 38.1 mm) were added, with nine replicates for each experimental group. These slides were used for subsequent bacterial density counting and larval metamorphosis experiments. After 48 h, the biofilms were removed from the culture dishes and fixed in 5% formalin solution for 24 h, followed by gentle rinsing with AFSW. The biofilms were stained with 0.1% acridine orange, dried, and subsequently observed and counted under a fluorescence microscope (Olympus BX51, Japan) for bacterial density calculation, using the calculation formula described by ref.^9^.

The ability of biofilm formation was determined by measuring bacterial density and thickness of biofilms. And the bacterial density was determined by CLSM analysis. Following the aforementioned biofilm cultivation and fixation steps, the biofilms were stained with 5 μg ml^−^^1^ propidium iodide^38^ for 15 min avoiding light exposure. Then excess dye on the biofilms was gently washed off with AFSW, and the slides were air-dried. Three randomly selected fields of view were observed on each of the three slides, using a Leica TCS SP8 CLSM, to examine bacterial distribution and biofilm thickness. The biofilms were separately stained with dyes for α-polysaccharide, β-polysaccharide, as well as proteins and lipids. Dye information is detailed in Supplementary Table 6. CLSM was utilized to capture images, and subsequent image analysis enabled the quantification of the content of each extracellular substance. The summation of these four substances was taken to represent the content of EPS. For detailed methods, refer to Liang et al.^66^.

### Determination of c-di-GMP content

We cultivated the biofilms and scraped them into double-distilled water to obtain 1 ml of OD600 = 1.5 solution (e.g., if the OD600 = 0.3, spin down 5 ml of solution).^78^ This solution was extracted using c-di-GMP extraction solution and collected for quantitative analysis using LC-MS/MS, with the method described by ref.^45^ A standard curve of c-di-GMP standard (Sigma, America) was used to calculate the concentration of the supernatant.

### Extraction, purification, and identification of OMVs

The extraction and purification of OMVs were performed following Wang et al.^35^. Sufficient amount of biofilm was scraped into physiological saline solution under ice-cold conditions to obtain a homogeneous suspension with the same OD value. OMVs were then extracted using ultracentrifugation and purified using sucrose density gradient centrifugation. The extracted OMVs were dissolved in PBS. The morphology of OMVs was observed with a HITACHI Regulus 8100 scanning electron microscope. To quantify the content of OMVs, the protein content within OMVs was used as a representative measure.^79^

### Acquisition of EPS and EPS-OMVs

Wild-type bacterial biofilm was cultured, dissolved in AFSW, and the supernatant was collected by centrifugation at 3500 rpm to remove bacterial precipitates, resulting in the isolation of EPS. The supernatant was further collected by high-speed centrifugation at 8000 rpm to remove sediment and obtain EPS-OMVs.

### Extraction and quantification of LPS

LPS from the bacterial cultures was extracted and quantified using the bacterial LPS extraction kit (X-Y Biotechnology, Shanghai) and an enzyme-linked immunosorbent assay (ELISA) detection kit (Mreda, Beijing), following the instructions provided. Standardization was carried out within the same experiment. For biofilm samples, twelve slides of the biofilm were combined as a single group. They were dissolved in 1 ml of double-distilled water for extraction and quantification, with four replicates per group. Subsequently, quantitative analysis was performed on the extracted LPS solution, with two replicates per tube. LPS content within OMVs was determined by extracting and quantifying LPS from 1 ml of OMV-PBS solution with a concentration of 1000 µg ml^−^^1^. Detection of major contaminants in LPS extraction samples was performed using the bicinchoninic acid assay (BCA assay) method for proteins and agarose gel electrophoresis for nucleic acids.^34,80^.

### Data analysis

Data were tested for normality using the Shapiro-Wilk W test. If the data conformed to a normal distribution, a Student’s t-test was performed. If the data did not conform to a normal distribution, non-parametric tests were conducted, and Wilcoxon tests were performed for each pair. Spearman’s rank correlation analysis was performed using JMP software, following the methodology described in ref.^39^.

### Reporting summary

Further information on research design is available in the Nature Research Reporting Summary linked to this article.

### Supplementary information

Supplementary Materials

Reporting summary

## Data Availability

The data analyzed in this study are available within the article and its supplementary files or from the corresponding author upon request.
