# Supplementary material for: Bacterial c-di-GMP signaling gene affects mussel larval metamorphosis through outer membrane vesicles and lipopolysaccharides
Source: NPJ Biofilms Microbiomes. 2024 Apr 4;10:38. doi: 10.1038/s41522-024-00508-6 (PMC10994910; doi:10.1038/s41522-024-00508-6)
Supplement: Supplementary file 1 — Supplementary Materials [file 41522_2024_508_MOESM1_ESM.pdf]

Supplementary Materials

Supplementary Table 1. Correlation analysis of OMV content with larval metamorphosis rate and c-di-GMP content

	Larval metamorphosis		c-di-GMP content	
	**r**	***p***	**r**	***p***
**OMV content**	**0.7295**	**0.0006*****	**0.7335**	**0.0019****

Supplementary Table 2. Correlation analysis of c-di-GMP content with LPS content of biofilms and OMVs

	LPS content of biofilms		LPS content of OMVs	
	r	*p*	r	*p*
C-di-GMP content	0.6357	0.0109*	0.6130	0.0151*

Supplementary Table 3. Correlation analysis of larval metamorphosis rate with LPS content of biofilms and OMVs

	LPS content of biofilms		LPS content of OMVs	
	**r**	***p***	**r**	***p***
**Larval metamorphosis**	**0.3912**	**0.0587**	**0.5643**	**0.0041****

Supplementary Table 4. Correlation analysis of larval metamorphosis rate with LPS content

	LPS content	
	**r**	***p***
**Larval metamorphosis**	**0.7545**	**0.0305***

Supplementary Table 5. Strains, plasmids and primers in this study

Strains or plasmids	Relevant characteristics	Source
**Strains**		
P. marina ECSMB14103	Wild-type	Ref 1.
E. coli WM3064	RP4(tra) in chromosome, DAP$^-$	Ref 2.
Δ*cdgB*	In-frame deletion of *cdgB*	This study
Δ*cdgB*::*cdgB*	Complementation of *cdgB*	This study
Δ*lps*	In-frame deletion of *lps*	This study
**Plasmids**		
pK18mobsacB-ery	pK18mobsacB containing the erythromycin resistant gene from pHT304, Kan^r, Ery^r	Ref 3.
pK18mobsacB-ery-*cdgB*	Recombinant plasmid for deleting *cdgB* genes	This study
pK18mobsacB-ery-*lps*	Recombinant plasmid for deleting *lps* genes	This study
pBBR1MCS-Cm	Broad-host-range vector containing the chloramphenicol resistant gene from pWD2	Ref 3.
PBBR1-Cm-*cdgB*	*cdgB* cloned into pBBR1MCS-Cm	This study
**Primers**		
**In-frame deletions**		
cdgB-up-F	CGCGGATCCTCGCCACGCTCGCATAC	
cdgB-up-R	CCGGAATTCAGATGATATTCTAAATAGTCATA	
cdgB-down-F	CCGGAATTCCTTGTAAACCTCTTAGTCTT	
cdgB-down-R	ACATGCATGCAGTGGACGGCGGTCAG	
cdgB-L-F	ACAGGGCTATGGTCGTAC	
cdgB-L-R	ACCCTTTGCTTGCTCATC	
cdgB-S-F	ATGAACGATGGATGTAGC	
cdgB-S-R	GGACTGTGATCTACTTCGTA	
lps-up-F	CGCGGATCCTATAGGGAATCTTTAGGC	
lps-up-R	CCGGAATTCTTTGGAACCTGCTTTCGG	
lps-down-F	CCGGAATTCATAAATCTCGGTTGATAG	
lps-down-R	GCTCTAGACCTCTTACAGTGAGTGGC	
lps-L-F	CAGGACCAGTGATTGAACG	
lps-L-R	AATGGCGATACGCTTTACG	
lps-S-F	CTTGATAGCCAAATACCGT	
lps-S-R	TTTACTCCCTCAGGCAGAT	
**Complementation**		
cdgB-pBBR-F	CCGGAATTCCTTGTAAACCTCTTAGTCTT	
cdgB-pBBR-R	ACGCGTCGACTATGACTATTTAGAATATCATCT	

Supplementary References

1. Peng, L.-H. et al. Complete genome of *Pseudoalteromonas marina* ECSMB14103, a mussel settlement-inducing bacterium isolated from the East China Sea. *Mar. Genomics.* **41**, 46-49 (2018).
2. Dehio, C. & Meyer, M. Maintenance of broad-host-range incompatibility group P and group Q plasmids and transposition of Tn5 in *Bartonella henselae* following conjugal plasmid transfer from *Escherichia coli*. *J. Bacteriol*. **179**, 538-540 (1997).
3. Wang, P. et al. Development of an efficient conjugation-based genetic manipulation system for *Pseudoalteromonas*. *Microb. Cell. Fact.* **14**, 1-11 (2015).

Supplementary Table 6. Dye used for confocal laser scanning microscopy and target

Dye	Target	Concentration	Detection wavelength
Propidium iodide (PI)	Dead cell	5 μg/mL	560-700 nm
Concanavalin A, tetramethylrhodamine conjugate (ConA-TMR)	α-polysaccharide	944.8 μg/mL	552-578 nm
Calcofluor white M2R (CFW)	β-polysaccharide	189 μg/mL	254-432 nm
DiIC18(5) oil, 1,1'-dioctadecyl-3,3,3',3'-tetramethylindodicarb-ocyanine perchlorate (DiD'oil)	Lipid	7.94 μg/mL	648-670 nm
Fluorescein isothiocyanate isomer I (FITC)	Protein	46.6 μg/Ml	495-519 nm

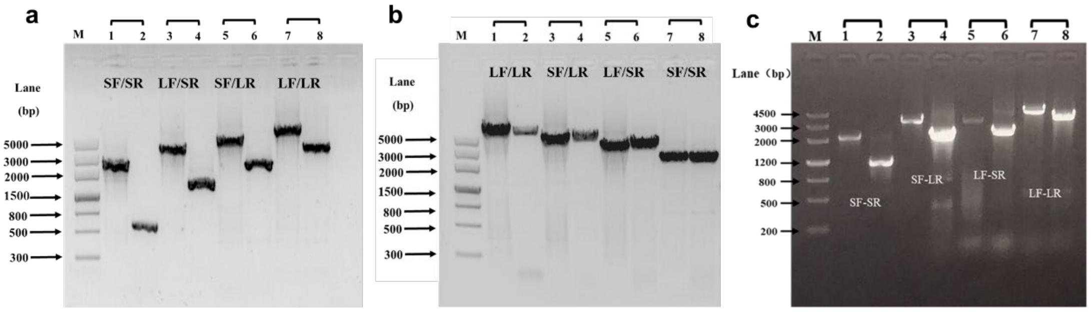

Supplementary Fig. 1. Identification of deleting (a) and complementary (b) the *cdgB* gene and deleting the *lps* gene (c) via PCR. Each panel depicts DNA bands derived from the same experiment, and they were processed in parallel.

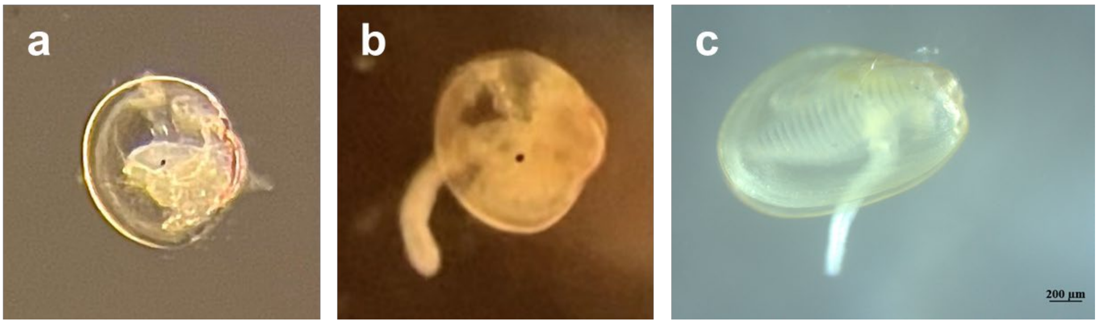

Supplementary Fig. 2. Larvae in stationary (a), crawling (b) state and Metamorphosed larvae (c).

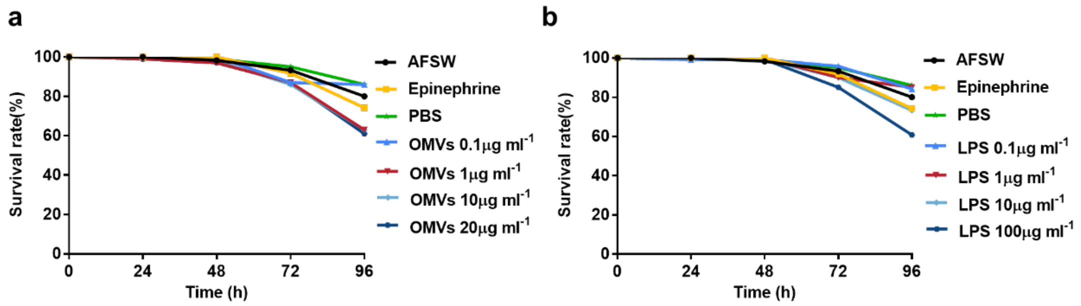

Supplementary Fig. 3. Survival rates during larval metamorphosis stimulated by OMVs (a) and LPS (b).
